# Inhomogeneous Distribution of Regional Myocardial Work Efficiency Predicts Early Left Ventricular Remodeling After Acute Anterior Myocardial Infarction Treated With Primary Percutaneous Intervention

**DOI:** 10.3389/fcvm.2022.922567

**Published:** 2022-07-27

**Authors:** Wei Wang, Hang Zhao, Fang Wan, Xue-dong Shen, Song Ding, Jun Pu

**Affiliations:** Department of Cardiology, Ren Ji Hospital, Shanghai Jiao Tong University School of Medicine, Shanghai, China

**Keywords:** ST-segment elevation myocardial infarction, myocardial work, work efficiency, left ventricular remodeling, echocardiography

## Abstract

**Background:**

This study aimed to investigate the hypothesis that specific pattern of myocardial work (MW) distribution in patients with acute anterior ST-segment elevation myocardial infarction (STEMI) could provide prognostic value for predicting left ventricular (LV) remodeling.

**Methods:**

A total of 98 first anterior wall STEMI patients treated with primary percutaneous coronary intervention [85 men (86.7%), mean age: 58 ± 12 years] were enrolled. Transthoracic echocardiography was performed 24–72 h after angioplasty and during 3-month follow-up. MW was estimated from the left ventricular pressure–strain loop derived from speckle tracking echocardiography and simultaneous noninvasive brachial artery cuff pressure. The primary endpoint was early LV remodeling, defined as an increase in LV end-diastolic volume ≥20% compared with baseline at 3 months after STEMI. Major adverse cardiac events and combined clinical outcomes were recorded.

**Results:**

LV remodeling was present in 32 patients (33%), who exhibited lower global and culprit-regional work index (WI), constructive work (CW), work efficiency (WE), and specifically, greater differences of WE (delta-WE) and CW (delta-CW) between the culprit and non-culprit region than those without LV remodeling both at the acute phase and follow-up (all *P* < 0.0125). During follow-up, all global and regional WI, CW, and WE were improved (*P* < 0.0125 compared with baseline), with less improvement in patients with LV remodeling. In multivariate analysis, baseline delta-WE (odds ratio: 2.304; 95% CI: 1.093–4.856, *P* = 0.028) and peak troponin I level (odds ratio: 1.035; 95%CI: 1.008–1.063, *P* = 0.010) were independently associated with early LV remodeling. Patients with greater delta-WE at baseline were associated with a higher incidence of heart failure and combined clinical outcomes during follow-up.

**Conclusion:**

After reperfused acute anterior STEMI, patients with LV remodeling presented with more inhomogeneous MW distribution. The absolute difference of WE between culprit and non-culprit territory at the acute phase is an independent predictor for early LV remodeling.

**Clinical Trial Registration:**

www.ClinicalTrials.gov, identifier: NCT05107102.

## Introduction

Despite successful reperfusion therapy and widespread prescription of anti-remodeling medications, left ventricular (LV) remodeling remains a relatively frequent event after acute myocardial infarction (MI) (1). Acute myocardial loss leads to a sudden increase in loading conditions, resulting in a unique remodeling pattern involving the infarct zone, infarct border zone, and the remote non-infarcted myocardium (2). Changes in LV structure occur 48–72 h after MI and may continue for weeks or months (3). Specifically, the incidence of remodeling was significantly higher in patients with anterior wall infarction compared with inferior or lateral infarction (3), which was associated with a higher risk of heart failure and increased mortality rate (4, 5). Therefore, it is intriguing to explore early predictors of LV remodeling in this subset.

Several mechanisms have been proposed underlying the pathology of cardiac remodeling (6). Recent studies (7, 8) suggested that inhomogeneities in myocardial workload played an important role in triggering the reverse remodeling process in dyssynchronous hearts. However, the assessment of myocardial work (MW) in clinical practice was limited due to the risk involving invasive measurement of LV pressure (9). Recently, a novel tool for noninvasive MW assessment has been introduced to evaluate myocardial performance (10, 11). This approach takes into account the impact of ventricular afterload and myocardial deformation by creating an LV pressure–strain loop, which integrated LV longitudinal strain derived from speckle-tracking echocardiography (STE) and noninvasively obtained brachial artery cuff pressure. The noninvasive pressure–strain loop has been verified to be related to invasive LV pressure measurements and can directly measure MW (10). A previous study confirmed that global MW was decreased in ischemic heart diseases (12, 13). However, the association between MW and post-infarct LV remodeling in patients with anterior MI has rarely been discussed (14, 15), and whether the inhomogeneous distribution of MW is associated with post-infarct remodeling is still unknown. In this study, we aimed to investigate the alteration of regional MW in patients with acute anterior MI who underwent primary percutaneous coronary intervention (PCI) and compare the distribution of regional MW in patients with/without early LV remodeling at the acute phase and 3-month follow-up. We hypothesized that a specific pattern of MW distribution at the acute phase may provide prognostic value for predicting cardiac remodeling.

## Methods

### Study Population

Patients with anterior ST-segment elevation myocardial infarction (STEMI) who underwent primary PCI < 12 h after symptom onset were consecutively enrolled in our center from September 2019 to May 2021. The diagnosis of acute anterior MI was based on chest pain lasting > 30 min, ST-segment elevation > 2 mm in at least two contiguous precordial electrocardiographic leads (from V1 to V6), and an increase in serum troponin I (16). In all patients, culprit vessel was left anterior descending coronary artery (LAD) with thrombolysis in myocardial infarction (TIMI) flow grade 0/1. Multivessel disease is defined as at least 1 additional non-culprit lesion, minimum with 2.5 mm in diameter and 70% diameter stenosis. Non-culprit lesion PCI was to be performed 30 ± 3 days after successful PCI of the culprit lesion. The exclusion criteria were as follows: significant valvular disease defined as moderate to severe valvular stenosis or regurgitation; cardiomyopathy; left bundle branch block; atrial fibrillation; previous MI; history of cardiac surgery; and pacemaker implantation. All patients were treated according to current guidelines for STEMI (16) and gave informed consent to the protocol. Information on medical history (hypertension, diabetes mellitus, and dyslipidemia) and current medication use were obtained. Laboratory and angiographic data were recorded. The Institutional Review Board of Renji Hospital approved this study, and all subjects provided written informed consent. The data that support the findings of this study are available on reasonable request to the corresponding author.

### Conventional Echocardiography

All subjects underwent conventional two-dimensional echocardiography (Vivid E95, GE Healthcare, Horton, Norway) at the acute phase (24–72 h after PCI) and at 3-month follow-up. Brachial pressure measurement was performed at the time of each examination. All echocardiographic examinations were performed by a senior cardiologist with subspecialty training in echocardiography. Apical two-, three-, and four-chamber views were stored at a frame rate of 50–70 Hz for offline analysis. Measurements were performed according to the recommended guidelines of the American Society of Echocardiography (17). Routine parameters included LV end-diastolic volume (LVEDV), LV end-systolic volume (LVESV), LV ejection fraction (LVEF), left atrial volume (LAV), early (E) and late (A) transmitral diastolic flow velocity, deceleration time of mitral E wave (DTE), an average of the septal and lateral annular mitral early diastolic (e) pulsed-wave tissue Doppler velocity, and the E/e ratio. LVEDV, LVESV, and LVEF were assessed in the apical four- and two-chambered views using the modified Simpson's method. LV remodeling was defined as an increase of the LV end-diastolic volume (LVEDV) ≥ 20% compared with baseline at 3 months after STEMI (2). LAV was indexed to body surface area (LAV index). A 16-segment model of the left ventricle was used for the wall motion score. The semiquantitative scoring system was described as follows: 1 = normal, 2 = hypokinesia, 3 = akinesia, and 4 = dyskinesia. Wall motion score index (WMSI) was derived as the sum of all scores divided by the number of segments visualized. All echocardiographic parameters were analyzed by investigators blinded to the clinical data.

### Myocardial Work and Global Longitudinal Strain Analysis

The LV global longitudinal strain (GLS) and MW indices were quantified using the vendor-specific module (EchoPAC version 203 software, GE Medical Systems, Horten, Norway). GLS was measured using two-dimensional speckle tracking echocardiography by manually tracing the LV endocardial border. The LS values from each of the 17 LV segments from four-, two-, and three-apical views were averaged to calculate LV GLS.

The MW was calculated as a function of time through a combination of LV GLS data and an estimated LV pressure curves (5). The peak LV pressure was substituted by the brachial cuff blood pressure taken immediately before the echocardiographic examination. A noninvasive LV pressure–strain curve was then constructed automatically by the software according to the blood pressure measurement and the duration of ejection and isovolumetric phases. The following variables were derived: global work index (GWI) was classified as total work within the area of the LV pressure–strain loop calculated from mitral valve closure to mitral valve opening; global constructive work (GCW) was defined as work contributing to LV ejection performed during shortening in systole and work performed during lengthening in isovolumic relaxation; global wasted work (GWW) was defined as work that not contribute to LV ejection performed during lengthening in systole and work performed during shortening in isovolumetric relaxation; and global work efficiency (GWE) was calculated from the ratio of the constructive work to the sum of constructive and wasted work, expressed as a percentage.

**Figure 1:**
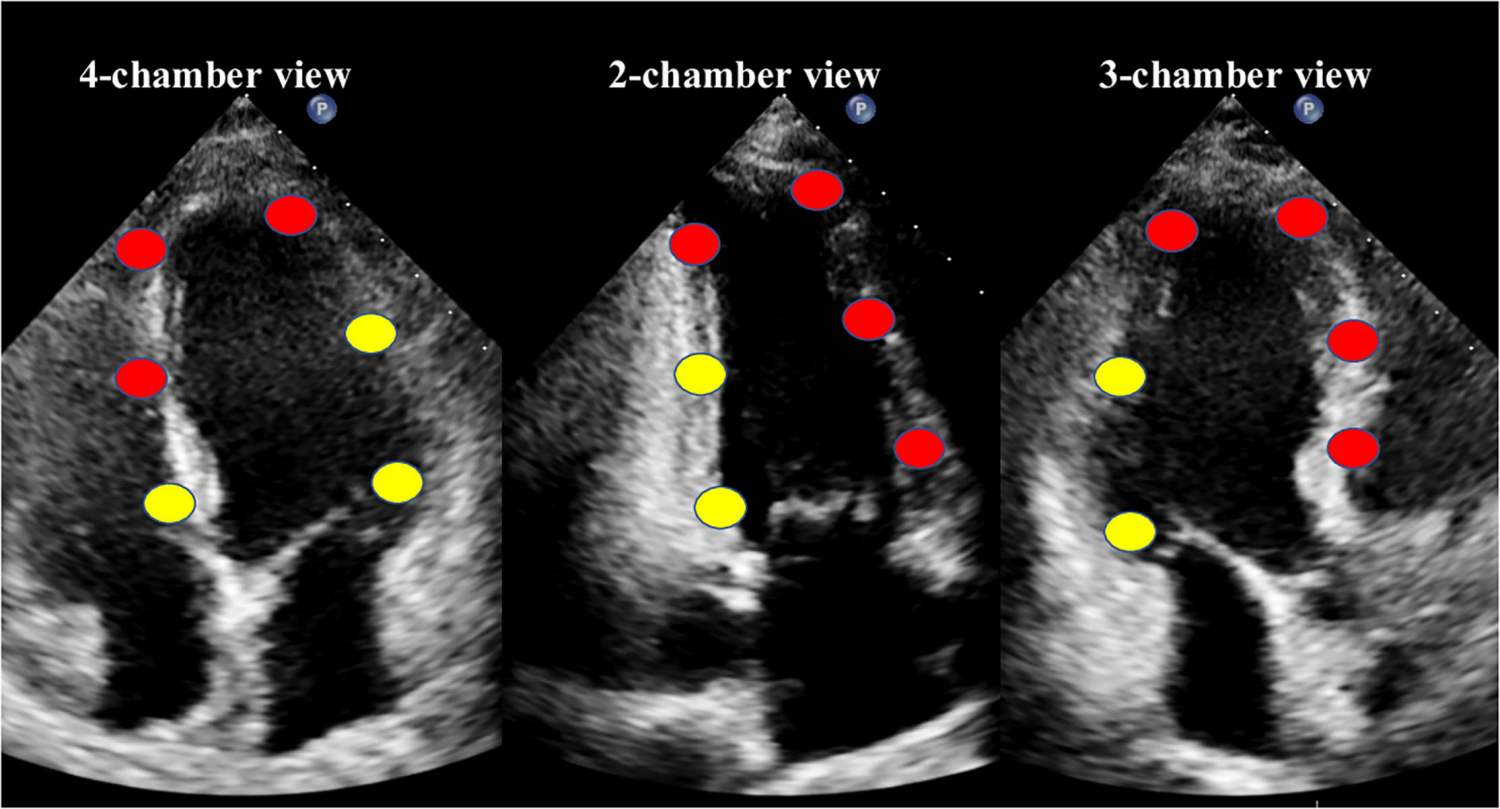
The18-segment model for myocardial work analysis. Red dots indicate the segments of the left anterior descending coronary artery (LAD) territory, while yellow dots indicate the non-LAD territory.

The values of culprit regional MW [including work index-LAD (WI-LAD), constructive work-LAD (CW-LAD), wasted work-LAD (WW-LAD), and work efficiency-LAD (WE-LAD)] and non-culprit regional MW (including WI-nonLAD, CW-nonLAD, WW-nonLAD, and WE-nonLAD) were obtained as the average of the segments belonging to the LAD or non-LAD territory (11 segments for the LAD, and 7 segments for the non-LAD; Figure 1) (18). The absolute differences of the MW indices were calculated as the values of non-culprit regional MW minus the values of culprit regional MW and noted as delta-WI, delta-CW, delta-WW, and delta-WE.

### Events

Major adverse cardiac events, consisting of cardiac death, hospitalization for heart failure, reinfarction, stroke, and combined clinical outcomes were recorded at 3 months. All-cause deaths will be considered cardiac unless a definite non-cardiac cause can be established. Hospitalization for heart failure was defined as prolonged hospitalization or rehospitalization due to clinical symptoms of congestive heart failure with clinical signs, including pulmonary edema, hypoperfusion or documented volume overload, and administration of intravenous diuresis or inotropic therapy. Reinfarction was defined as recurrent chest pain associated with new electrocardiographic changes and a significant increase of serum cardiac enzymes or troponin. Stroke was defined as either ischemic or hemorrhagic stroke showing a new focal neurologic deficit thought to be vascular in origin, with signs or symptoms lasting more than 24 h.

### Statistical Analysis

Sample size calculation was performed using power analysis and sample size (PASS) software (version 11.0.7; PASS, NCSS, LLC). According to previous studies, the rate of LV remodeling after anterior MI was approximately 28–38% (19, 20), we expect to include twice as many no LV remodeling cases than LV remodeling cases (the ratio of sample sizes in negative/positive groups = 2). Based on our pretest, the expected area under the receiver-operating characteristic (ROC) curve was at least 0.70, assuming α = 0.05, β = 0.15 (power = 0.85), requiring at least 27 cases in the LV remodeling group and 54 cases in the no LV remodeling group. Assuming a 10% loss to follow-up rate, a total of 90 subjects were required.

Statistical analysis was performed using SPSS (version 23; IBM, Chicago, IL) and MedCalc (version 15.2.0; MedCalc, Mariakerke, Belgium). The Kolmogorov–Smirnov test was used to examine the normality of distribution. Continuous variables were expressed as mean ± SD (if normally distributed) or median and interquartile ranges (if not normally distributed). Categorical variables were expressed as percentages. To compare the values between patients with and without LV remodeling, independent *t*-tests for a nonnormal distribution and nonparametric tests, such as Mann–Whitney U test for a nonnormal distribution, were performed to assess differences in unpaired samples. Fisher's exact test was used for categorical data. To compare the changes in each group, paired *t*-tests or Wilcoxon tests were used. Logistic regression models were performed to test independent correlates of LV remodeling. Clinical characteristics and baseline echocardiographic parameters were included in the univariate analysis. To avoid the collinearity problems, age, sex, and variables with *P* < 0.05 in univariate analysis were included in a stepwise multivariate regression model (LR Forward). ROC curve analysis and Youden index were assessed to determine optimal cutoff points for sensitivity and specificity for identifying patients with LV remodeling. Intra- and interobserver variability of MW parameters was assessed using the intraclass correlation coefficient (ICC). A total of 20 subjects were randomly selected for that analysis. A two-tailed *P* < 0.05 was considered statistically significant. Furthermore, threshold level of significance for differences between groups was adjusted for multiple comparisons by Bonferroni's correction. The differences were statistically significant when the observed *P*-values were less than the specified significance level (α) divided by the number of tests (*K*) = 0.05/4.

## Results

### Baseline Characteristics of the Study Population

**Figure 2:**
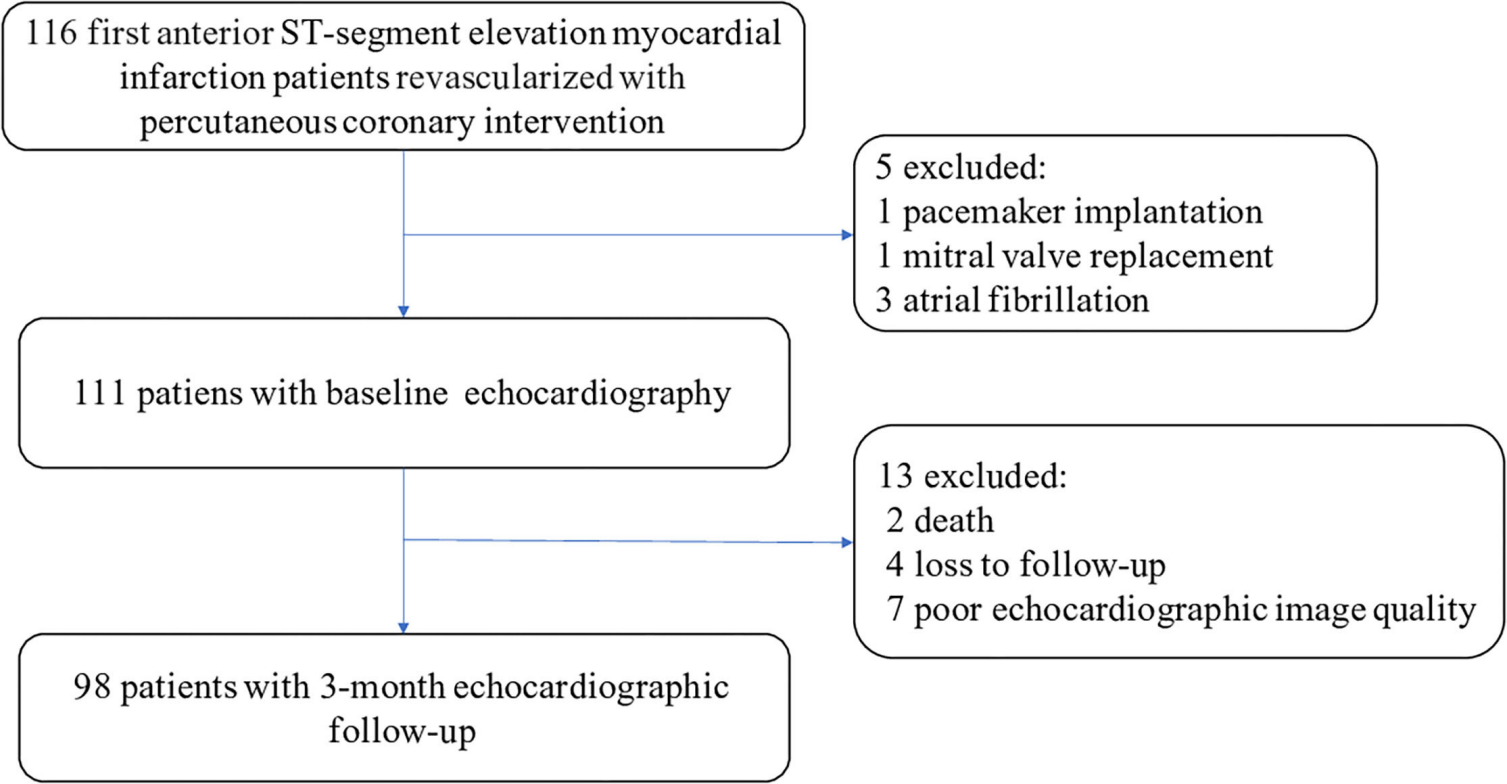
Flowchart for the selection of our study population.

A total of 116 patients with anterior MI were initially identified. Of them, 18 patients were further excluded: one patient had a history of pacemaker implantation, one patient had a history of mitral valve replacement, three patients had atrial fibrillation, two patients died during follow-up, four patients were not available for the follow-up at 3-month, and seven patients had poor echocardiographic quality images (Figure 2). A total of 98 patients [85 men (87%), mean age: 58 ± 12 years] were ultimately included in our study for image analysis. Based on the echocardiographic criteria, LV remodeling was present in 32 patients (33%). Comparisons of baseline clinical characteristics and conventional echocardiographic parameters of patients with and without LV remodeling are summarized in Tables 1, 2. Patients with LV remodeling were associated with higher level of peak troponin I (TNI) (60.91 [range, 27.86–83.00] ng/ml vs. 27.86 [range, 13.52–56.59] ng/ml; *P* = 0.001) and BNP (426 [range, 194–1,084] pg/ml vs. 231 [range, 118–460] pg/ml; *P* = 0.029) level, as well as lower percentage of final TIMI 3 flow grades (81 vs. 96%, *P* = 0.022). For echocardiographic parameters, patients with LV remodeling exhibited reduced DTE (120 [range, 109–142] ms vs. 133 [range, 114–160] ms; *P* = 0.040), lower LVEF (44 ± 7% vs. 51 ± 7%; *P* < 0.001), higher WMSI (1.75 [range, 1.63–2.00] vs. 1.63 [range, 1.38–1.75]; *P* = 0.019), and significantly impaired LV GLS (−9 [range, −10–−8] % vs. −11 [range, −13–−9] %; *P* < 0.001) than those without LV remodeling.

**Table 1:** Baseline characteristics of the study population.

**Clinical characteristics**	**All patients**	**No LV remodeling**	**LV remodeling**	***P***
	**(*n* = 98)**	**(*n* = 66)**	**(*n* = 32)**	
Age, years	58 ± 12	59 ± 11	56 ± 13	0.243
Male, *n* (%)	85 (87)	56 (85)	29 (91)	0.429
Body mass index, kg/m^2^	25.13 ± 3.03	25.26 ± 3.07	24.89 ± 2.98	0.584
Hypertension, *n* (%)	50 (51)	35 (53)	15 (47)	0.568
Diabetes, *n* (%)	25 (26)	19 (29)	6 (19)	0.285
Dyslipidemia, *n* (%)	33 (34)	20 (30)	13 (41)	0.311
eGFR ≤ 60 ml/min/1.73^2^	4 (4)	2 (3)	2 (6)	0.450
Systolic blood pressure, mmHg	125 ± 19	126 ± 17	122 ± 21	0.241
Diastolic blood pressure, mmHg	78 ± 12	78 ± 12	78 ± 14	0.897
Heart rate, bpm	80 (72–85)	80 (70–84)	82 (75–88)	0.151
Peak troponin I, ng/ml	27.86 (16.75–78.00)	27.86 (13.52–56.59)	60.91 (27.86–83.00)	**0.001**
Peak creatine phosphokinase, U/L	1,600 (795–4,401)	1,522 (696–3,559)	2,571 (1,062–6,780)	0.122
BNP, pg/ml	272 (136–654)	231 (118–460)	426 (194–1,084)	**0.029**
Multi-vessel disease, n (%)	32 (33)	24 (36)	8 (25)	0.261
Symptom onset to balloon time (min)	267 (177–392)	264 (175–374)	283 (195–440)	0.418
Final TIMI 3 flow grade, *n* (%)	89 (91)	63 (96)	26 (81)	**0.022**
Complete revascularization, *n* (%)	26 (81)	19 (79)	7 (88)	0.601
Dual-antiplatelet therapy, *n* (%)	98 (100)	66 (100)	32 (100)	-
Statin, *n* (%)	94 (96)	63 (96)	31 (97)	0.739
ACEI/ARB/ARNI, *n* (%)	86 (88)	60 (91)	26 (81)	0.171
Beta-blocker, n (%)	93 (95)	63 (96)	30 (94)	0.719

*Values are expressed as number (percentage), or mean ± standard deviation, or median (interquartile range) depending on data normality. ACEI, angiotensin-converting enzyme inhibitor; ARB, angiotensin II receptor blocker; ARNI, angiotensin-receptor neprilysin inhibitor; LV, left ventricular; TIMI, thrombolysis in myocardial infarction. Significant P values are in boldface type*.

**Table 2:** Baseline echocardiographic characteristics of patients with or without LV remodeling.

	**No LV remodeling**	**LV remodeling**	***P***
	**(*n* = 66)**	**(*n* = 32)**	
Left atrial volume index, ml/m^2^	20 ± 5	21 ± 5	0.986
LVEDV, ml	91 ± 20	87 ± 14	0.291
LVESV, ml	45 ± 13	49 ± 11	0.179
Mitral E/A ratio	0.8 (0.7–1.2)	0.7 (0.6–1.0)	0.276
DTE, msec	133 (114–160)	120 (109–142)	**0.040**
E/e' ratio^*^	8.4 (7.0–10.4)	8.4 (7.2–10.7)	0.969
LVEF, %	51 ± 7	44 ± 7	**<0.001**
WMSI	1.63 (1.38–1.75)	1.75 (1.63–2.00)	**0.019**
GLS, -%	11 (9–13)	9 (8–10)	**<0.001**

*Values are expressed as mean ± standard deviation, or median (interquartile range). DTE, deceleration time of mitral E wave; EDV, end-diastolic volume; EF, ejection fraction; ESV, end-systolic volume; LV, left ventricular; GLS, global longitudinal strain; WMSI, wall motion score index;*

* *Average of the septal and lateral annular mitral early diastolic tissue Doppler velocity. Significant P values are in boldface type*.

### Comparison of MW Between Patients With and Without LV Remodeling at the Acute Phase

**Figure 3:**
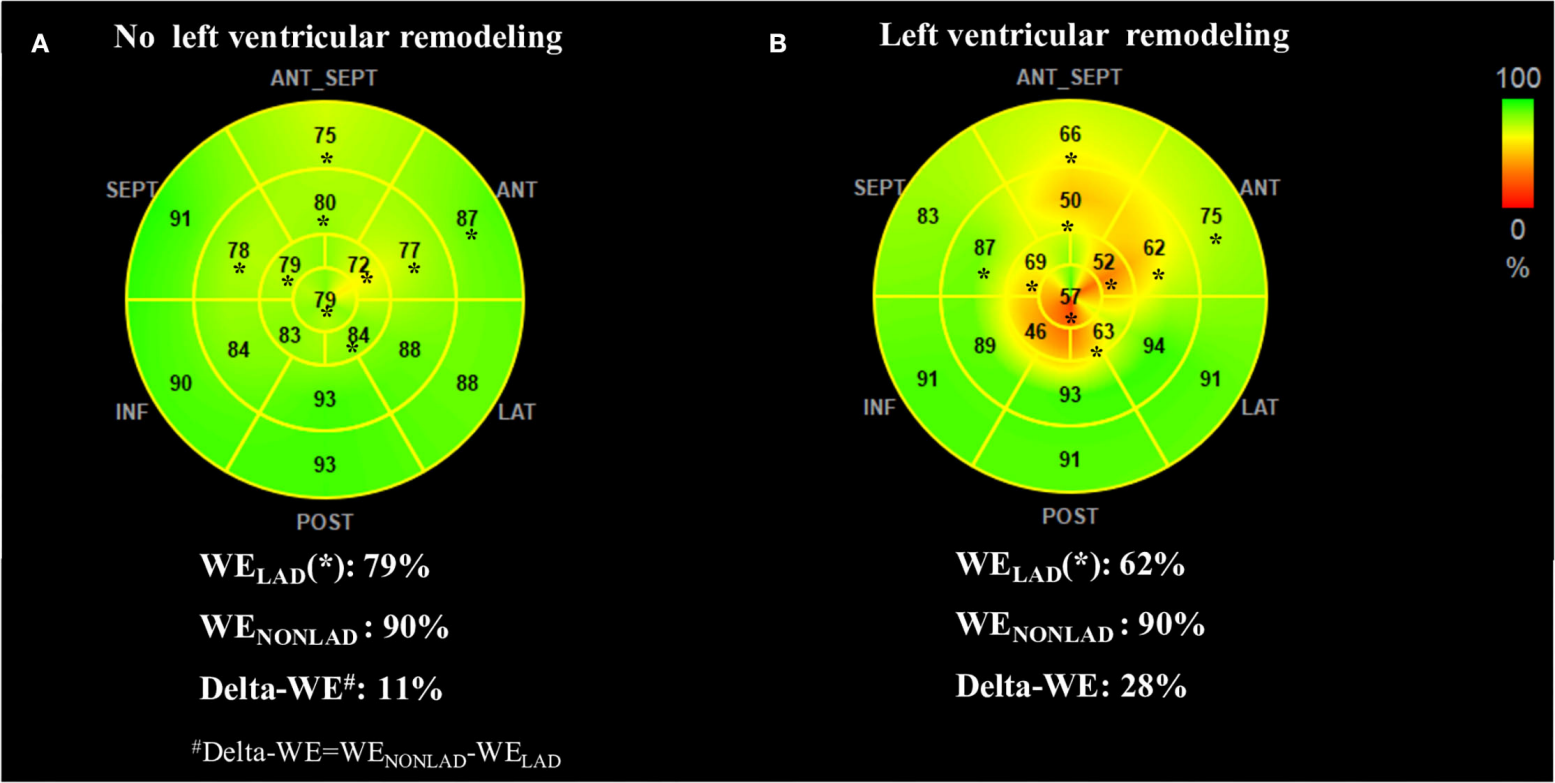
Representative WE bull's-eye plots of patients without **(A)** and with **(B)** early left ventricular remodeling. Asterisks indicate segments assigned to the left anterior descending coronary artery (LAD) territory. The no-remodeling patient showed relatively homogeneous regional WE (presented in green) between LAD and non-LAD territory. In contrast, the remodeling patient exhibited significantly reduced WE in LAD territory (presented in yellow and orange). The index of delta-WE (calculated as WE_NONLAD_ – WE_LAD_) was much greater in the remodeling patient than the no-remodeling (28 vs. 11%), indicating a more inhomogeneous distribution of regional WE. LAD, left anterior descending artery coronary artery; LV, left ventricular; WE, work efficiency; WE_LAD_, average of segmental WE in LAD territory; WE_NONLAD_, average of segmental WE in non-LAD territory.

MW indices at the acute phase are presented in Table 3. The patients with LV remodeling presented with lower values of GWI (763 ± 206 mmHg% vs. 927 ± 254 mmHg%, *P* = 0.002), GWE (78 [range, 74–82] % vs. 84 [range,79–89] %; *P* < 0.001), and GCW (970 ± 229 mmHg% vs. 1,179 ± 261 mmHg%, *P* < 0.001) compared with those without LV remodeling. Similarly, significantly reduced regional WI-LAD (671 ± 242 mmHg% vs. 864 ± 280 mmHg%, *P* = 0.001), WE-LAD, (71% ± 9% vs. 80% ± 8%, *P* < 0.001) and CW-LAD (667 [range, 530–911] mmHg% vs. 985 [range, 807–1,183] mmHg%; *P* < 0.001) were observed in the LV remodeling group, while there was no difference for MW indices in the non-culprit territory between the two groups. Although the values of GWW (227 ± 75 mmHg% vs. 189 ± 75 mmHg%, *P* = 0.022) and WW-LAD (264 ± 105 mmHg% vs. 214 ± 92 mmHg%, *P* = 0.018) had an increased tendency in the LV remodeling group than the group without LV remodeling, there were no significant differences between the two groups. Of note, the difference of regional WE and regional CW between the culprit territory and the non-culprit territory at acute phase were higher in patients with LV remodeling compared with that in patients without LV remodeling, as indicated by higher delta-WE (20 [range, 11–26] % vs. 9 [range, 3–15] %; *P* < 0.001) and delta-CW (587 ± 262 mmHg% vs. 425 ± 245 mmHg%, *P* < 0.004). Figure 3 shows the representative bull's-eye plots for regional WE distribution of patients with and without early LV remodeling.

**Table 3:** Comparison of MW between two groups at acute phase and follow-up.

	**No LV remodeling (*****n*** **=** **66)**		**LV remodeling (*****n*** **=** **32)**			***P*** **-Value**		
	**Acute Phase**	**Follow-Up**	**Acute Phase**	**Follow-Up**	***P①***	***P②***	***P③***	***P④***
**Global MW indices**								
GWI (mmHg%)	927 ± 254	1,254 ± 367^*^	763 ± 206^#^	977 ± 305^*^^#^	**<0.001**	**0.001**	**0.002**	**<0.001**
GWE (%)	84 (79–89)	89 (84–93)^*^	78 (74–82)^#^	81 (78–89)^*^^#^	**<0.001**	**0.003**	**<0.001**	**0.001**
GCW (mmHg%)	1179 ± 261	1542 ± 390^*^	970 ± 229^#^	1241 ± 353^*^^#^	**<0.001**	**<0.001**	**<0.001**	**<0.001**
GWW (mmHg%)	189 ± 75	175 ± 81	227 ± 75	207 ± 98	0.182	0.225	0.022	0.083
**Regional MW indices in culprit territory**								
WI-LAD (mmHg%)	864 ± 280	1,197 ± 432^*^	671 ± 242^#^	844 ± 352^*^^#^	**<0.001**	**0.007**	**0.001**	**<0.001**
WE-LAD (%)	80 ± 8	85 ± 8^*^	71 ± 9^#^	76 ± 11^#^	**<0.001**	0.014	**<0.001**	**<0.001**
CW-LAD (mmHg%)	985 (807–1,183)	1,361 (1,001–1,652)^*^	667 (530–911)^#^	995 (662–1,249)^*^^#^	**<0.001**	**0.001**	**<0.001**	**<0.001**
WW-LAD (mmHg%)	214 ± 92	198 ± 95	264 ± 105	250 ± 124	0.247	0.519	0.018	0.023
**Regional MW indices in non-culprit territory**								
WI-nonLAD (mmHg%)	1,025 ± 271	1,341 ± 316 ^*^	923 ± 233	1,191 ± 294^*^	**<0.001**	**<0.001**	0.069	0.026
WE-nonLAD (%)	90 (86–94)	93 (90–95)^*^	89 (86–92)	92 (89–95)^*^	**0.001**	**0.001**	0.144	0.540
CW-nonLAD (mmHg%)	1,421 ± 294	1,778 ± 350^*^	1,328 ± 275	1,638 ± 351^*^	**<0.001**	**<0.001**	0.141	0.066
WW-nonLAD (mmHg%)	132 (76–210)	110 (73–184)	171 (108–222)	128 (68–191)	0.425	0.061	0.166	0.985
**Absolute differences of the MW indices**								
Delta-WI (mmHg%)	232 ± 149	263 ± 177	293 ± 195	366 ± 221	0.197	0.094	0.121	0.015
Delta-WE (%)	9 (3–15)	6 (3–11)	20 (11–26)^#^	17 (8–22)^#^	0.046	0.072	**<0.001**	**<0.001**
Delta-CW (mmHg%)	425 ± 245	458 ± 285	587 ± 262^#^	650 ± 247^#^	0.413	0.225	**0.004**	**0.002**
Delta-WW (mmHg%)	59 (32–126)	68 (28–113)	84 (53–181)	128 (47–183)	0.779	0.519	0.231	0.017

*MW, myocardial work; LV, left ventricular; GWI, global work index; GCW, global constructive work; GWW, global wasted work; GWE, global work efficiency; LAD, left anterior descending coronary artery; delta-WI, delta-CW, delta-WW, and delta-WE were defined as the absolute differences of the MW indices between non-LAD territory and LAD territory. P① no LV remodeling at follow-up vs. no LV remodeling at acute phase; P② LV remodeling at follow-up vs. LV remodeling at acute phase; P③ LV remodeling at acute phase vs. no LV remodeling at acute phase; P④ LV remodeling at follow-up vs. no LV remodeling at follow-up;*

* *P < 0.0125 (0.05/4) vs. acute phase;*

# *P < 0.0125 (0.05/4) vs. no LV remodeling*.

*Significant P values are in boldface type*.

### Comparison of MW Between the Acute Phase and Follow-Up

Evolution of MWs between the acute phase and 3-month follow-up is shown in Table 3. There was a significant improvement of GCW, GWI, and GWE at follow-up in both groups with and without LV remodeling (all *P* < 0.0125). For regional MW indices, an improvement of regional WI, WE, and CW were presented not only in LAD territory but also in the non-LAD territory in patients with or without LV remodeling at follow-up compared with the acute phase (nearly all *P* < 0.0125). In contrast, no improvement of global or regional WW was observed (all *P* > 0.0125). Of note, despite the recovery of MW, patients with LV remodeling still exhibited impaired global and culprit-regional MW indices (except GWW and WW-LAD), as well as greater delta-WE and delta-CW compared with patients without LV remodeling at follow-up (all *P* < 0.0125).

### Clinical and Echocardiographic Parameters Predicting LV Remodeling

**Figure 4:**
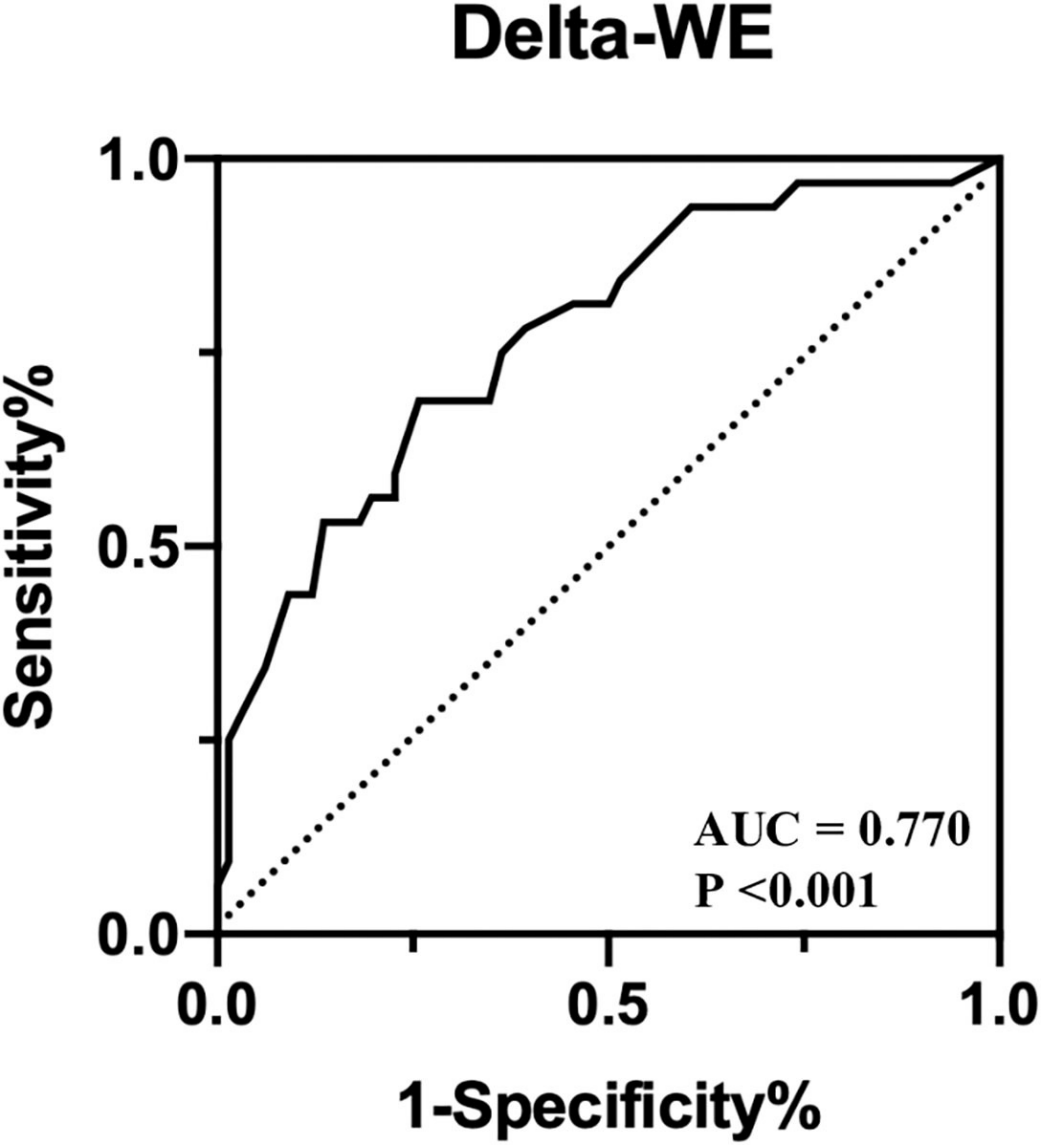
Receiver operating characteristic curves of delta-WE to identify early LV remodeling in anterior STEMI patients.

**Figure 5:**
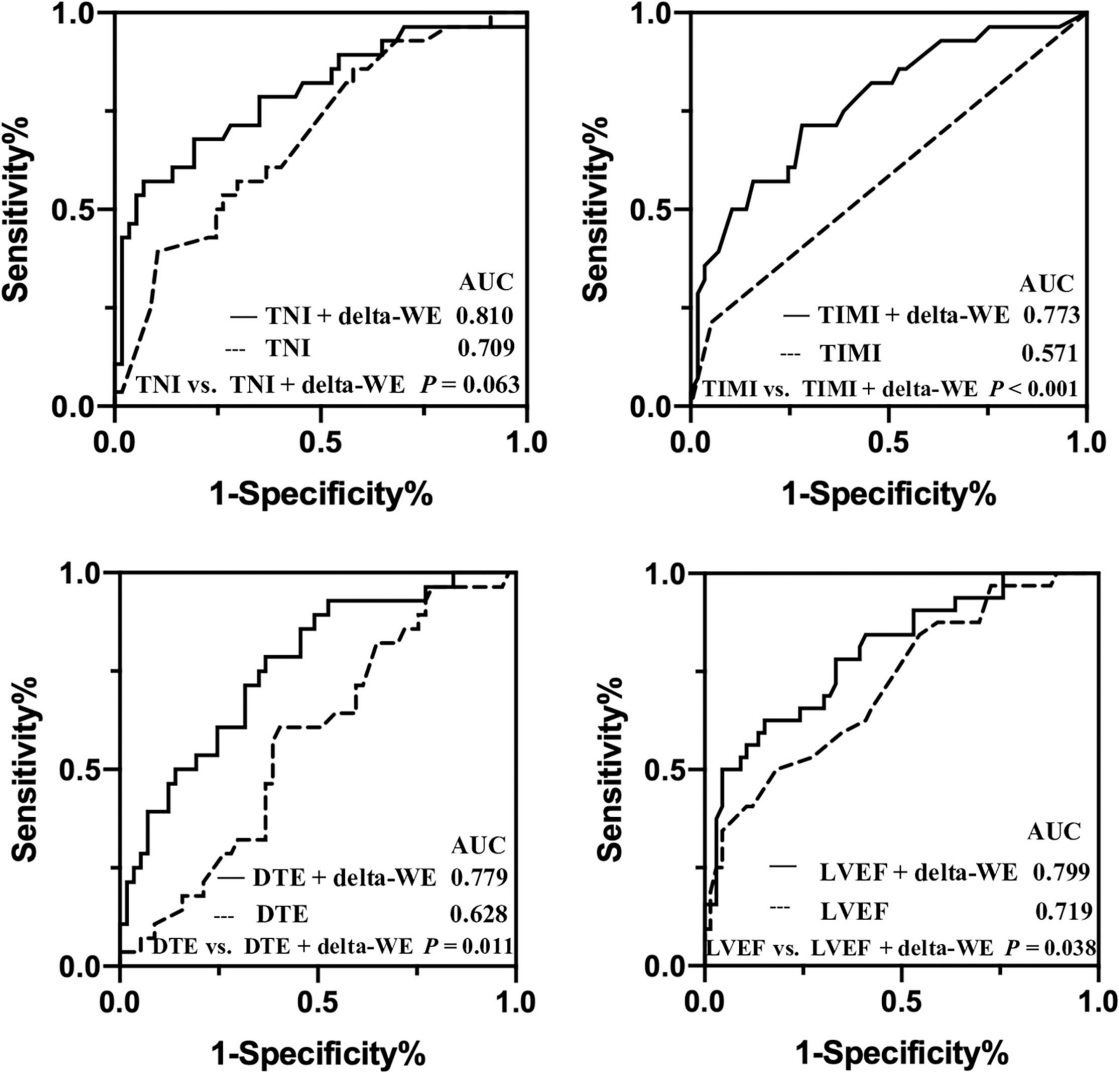
Comparisons of receiver operating characteristic curves of baseline traditional clinical risk factors with and without delta-WE to predict early left ventricular remodeling in anterior STEMI patients. AUC, area under the curve.

Univariate and multivariate regression analyses for identifying predictors of early LV remodeling are presented in Table 4. Age, gender, and variables with *P* < 0.05 in the univariate regression (including TNI, final TIMI 3 flow grade, DTE, LVEF, WMSI, GLS, and MW indices) were included in a stepwise multivariate regression model (LR Forward), which identified that only TNI and delta-WE were independently associated with the incidence of early LV remodeling. Of note, each 1% increase in delta-WE was associated with a 2.30-fold increased risk of LV remodeling (adjusted odds ratio: 2.304; 95% CI: 1.093–4.856, *P* = 0.028). The ROC analysis revealed a cutoff value of ≥15% for delta-WE to predict early LV remodeling with a sensitivity of 69% and specificity of 74% (AUC = 0.770, *P* < 0.001, Figure 4). Comparisons of ROC curves showed that models integrating delta-WE significantly improved the diagnostic value of traditional clinical and echocardiographic parameters (including TNI, final TIMI 3 flow grade, DTE, and LVEF) to predict LV remodeling (Figure 5, Table 5).

**Table 4:** Uni- and multivariate logistic regression analysis for identifying predictors of early LV remodeling.

	**Univariate**			**Multivariate**		
	**Odds ratio**	**95% confidence interval**	***P***	**Odds ratio**	**95% confidence interval**	***P***
Age (years)	0.978	0.942–1.015	0.241			
Sex (female)	1.726	0.440–6.765	0.433			
TNI (ng/ml)	1.024	1.008–1.040	**0.003**	1.035	1.008–1.063	**0.010**
Final TIMI 3 flow grade	4.846	1.126–20.852	**0.034**			
DTE (msec)	0.983	0.969–0.999	**0.033**			
LVEF (%)	0.877	0.814–0.945	**0.001**			
WMSI	5.006	1.157–21.649	**0.031**			
GLS (-%)	1.578	1.225–2.032	**<0.001**			
GWI (mmHg%)	0.997	0.995–0.999	**0.004**			
GWE (%)	0.818	0.743–0.902	**<0.001**			
GCW (mmHg%)	0.996	0.994–0.998	**<0.001**			
GWW (mmHg%)	1.007	1.001–1.013	**0.024**			
WI-LAD (mmHg%)	0.997	0.995–0.999	**0.002**			
WE-LAD (mmHg%)	0.874	0.821–0.931	**<0.001**			
CW-LAD (mmHg%)	0.996	0.994–0.998	**<0.001**			
WW-LAD (mmHg%)	1.005	1.001–1.010	**0.021**			
Delta-WE (mmHg%)	1.129	1.065–1.198	**<0.001**	2.304	1.093–4.856	**0.028**
Delta-CW (mmHg%)	1.003	1.001–1.004	**0.005**			

*Age, sex, and variables with a P < 0.05 in the univariate analysis (bold) were included in a stepwise multiple regression model (LR Forward). Variables with P > 0.05 in univariate analysis were not displayed in the table. Abbreviations are same as in Tables 1–3*.

**Table 5:** Receiver operating characteristic analysis of different models for predicting early left ventricular remodeling in anterior STEMI patients.

	**AUC**	**Sensitivity** **(%)**	**Specificity (%)**	**Youden index**	**Cut-off value**	***P***
Delta-WE (mmHg%)	0.770	69	74	0.43	≥15	<0.001
TNI (ng/ml)	0.709	43	90	0.33	>78	<0.001
TNI+delta-WE	0.810	60	93	0.53		<0.001
Final TIMI 3 flow grade	0.571	19	95	0.14	≤ 2	0.242
Final TIMI 3 flow grade+delta-WE	0.773	56	86	0.42		<0.001
DTE (msec)	0.628	66	64	0.3	≤ 123	0.028
DTE+delta-WE	0.779	94	50	0.44		<0.001
LVEF (%)	0.719	50	82	0.32	≤ 44	<0.001
LVEF+delta-WE	0.799	63	85	0.48		<0.001

*Abbreviations are same as in Tables 1–3. AUC, Area under the curve*.

### Events

To analyze the relationship between delta-WE and adverse events, we added the two death cases to the 98-case cohort. Major adverse cardiac events occurred in 14 (14%) patients, including 2 (2%) cardiac death, 11 (11%) heart failure, and 1 (1%) reinfarction. There was no stroke occurred during follow-up. When the cutoff value of ≥15% for delta-WE was applied, we observed higher incidence of heart failure and combined clinical outcomes in patients with delta-WE ≥15% compared with those with delta-WE <15% (*P* = 0.019 and 0.010, respectively). There was no significant difference in the rate of cardiac death and reinfarction between the two subgroups (*P* = 0.771 and 0.218, respectively) (Table 6).

**Table 6:** Major adverse cardiac events at 3 months.

**Clinical outcomes**	**Delta-WE ≥15%**	**Delta-WE <15%**	**Odds Ratio**	***P***
	**(*n* = 40)**	**(*n* = 60)**	**(95% CI)**	
Cardiac death, *n* (%)	1 (2.5)	1 (1.7)	1.513 [0.092–24.907]	0.771
Hospitalization for heart failure, *n* (%)	8 (20.0)	3 (5.0)	4.750 [1.176–19.180]	**0.019**
Reinfarction, *n* (%)	1 (2.5)	0 (0)	1.026 [0.976–1.078]	0.218
Stroke, *n* (%)	0 (0)	0 (0)	-	–
Combined clinical outcomes, *n* (%)	10 (25.0)	4 (6.7)	4.667 [1.349–16.149]	**0.010**

*Abbreviations are same as in Table 3. Significant P values are in boldface type*.

### Reproducibility

Intra- and interobserver agreement was strong for both global and regional MW indices and is shown in Table 7.

**Table 7:** ICCs for intra- and interobserver variability for MW indices.

**Variable**	**Interobserver variability**		**Intraobeserver variability**	
	**ICC**	**95% confidence interval**	**ICC**	**95% confidence interval**
GWI (mmHg%)	0.980	0.960–0.990	0.989	0.978–0.995
GWE (%)	0.949	0.898–0.975	0.965	0.929–0.981
GCW (mmHg%)	0.982	0.963–0.991	0.985	0.970–0.993
GWW (mmHg%)	0.939	0.878–0.970	0.940	0.877–0.971
WI-LAD (mmHg%)	0.945	0.889–0.973	0.968	0.935–0.984
WE-LAD (%)	0.943	0.885–0.972	0.991	0.982–0.996
CW-LAD (mmHg%)	0.983	0.965–0.992	0.985	0.969–0.993
WW-LAD (mmHg%)	0.920	0.840–0.960	0.926	0.849–0.964
WI-nonLAD (mmHg%)	0.955	0.908–0.978	0.965	0.929–0.983
WE-nonLAD (%)	0.868	0.746–0.934	0.804	0.634–0.901
CW-nonLAD (mmHg%)	0.960	0.918–0.980	0.886	0.777–0.944
WW-nonLAD (mmHg%)	0.890	0.785–0.945	0.884	0.773–0.942

*ICC, intraclass correlation coefficient. Abbreviations are same as in Table 3*.

## Discussion

The main findings of our study could be summarized as follows. (1) After an acute anterior MI, patients with early LV remodeling had more profound impairment of global and culprit-regional MW both at the acute phase and 3-month follow-up compared with those without LV remodeling. (2) During follow-up, MW indices (except for WW) were improved in both culprit and non-culprit region, with less improvement of MWs in patients with LV remodeling in comparison with those without remodeling. (3) Among MW indices at acute phase, the absolute difference of WE between culprit and non-culprit territory (delta-WE) was independently associated with the incidence of early LV remodeling, suggesting the imbalanced distribution of myocardial work efficiency after MI might serve as an independent predictor of LV remodeling.

### Comparison With Previous Studies

Several studies have investigated the alteration of MW in coronary heart disease. Compared with strain parameters derived from STE, MW assessment had exhibited higher sensitivity and specificity in identifying severe coronary stenosis or occlusion, as it took into account both myocardial deformation and the impact of afterload (12, 21). Meimoun et al. (13) explored the prognostic value of MW in MI and proposed that myocardial constructive work was superior to longitudinal strain and other MW indices in predicting global and segmental functional recovery at 3-month follow-up. However, the association between MW and post-infarct LV remodeling has rarely been discussed. Lustosa et al. (14) demonstrated that regional MW in the culprit territory in acute phase was a powerful predictor of early LV remodeling, however, the study cohort constituted patients with different culprit vessels. It was noted that in this study, patients with anterior MI exhibited more impaired baseline MW both in the culprit and non-culprit territory compared with non-anterior MI and the incidence of remodeling was significantly higher in this subset, suggesting that the cutoff value and diagnostic accuracy of MWs for LV remodeling might vary in subgroups with the different target vessel. Therefore, we conducted this study specifically addressing the predictive value of MW parameters in anterior MI.

### Evolution of Regional MW After MI

Consistent with previous study (14), we observed that MW in culprit territory was significantly impaired at the acute phase of MI compared with non-culprit territory despite timely reperfusion therapy, and we further explore the evolution of regional MW during follow-up. We found the improvement of regional WI, CM, and WE not only occurred in the culprit territory but also in non-culprit territory. Our result was in agreement with previous cardiac magnetic resonance (CMR) study (22), which revealed the presence of myocardial dysfunction in the remote, non-culprit territory in acute phase of anterior MI. The potential mechanisms underlying dysfunction in non-culprit territory could be attributed to the impact of morphologic changes in the infarct-related myocardium on myocardial deformation (22), or the myocardial ischemia and metabolic disorders due to diffused coronary artery spasm and endothelial dysfunction during acute attack of ischemia (23). Our findings supported that myocardial dysfunction in non-culprit territory also contributed to global dysfunction in the acute phase of MI. In contrast, there was no significant improvement of global or regional WW at follow-up compared with baseline. Increased WW has been recognized as an indicator for uncoordinated systolic motion, including both post-systolic shortening and lengthening during systolic phase. Our finding suggested the persisted systolic dyssynchrony after MI due to the inhomogeneity of regional myocardial contractility and conduction (24).

### Inhomogeneous Regional MW Distribution and LV Remodeling

Compared with patients without LV remodeling, patients with LV remodeling were associated with more profound impairment of global and culprit-regional MW at acute phase, along with higher TNI level, which may be associated with larger infarct size. Meanwhile, greater difference of WE between culprit and non-culprit territory was observed in patients with LV remodeling indicating a more inhomogeneous distribution of MW at the beginning of remodeling period, which was also observed in dyssynchronous hearts. Previous studies (8) in animal model with pacemaker induced dyssynchrony suggested that cardiac remodeling starts as an adaptive response toward the equilibration of regional MW distribution, during which the segments with reduced workload and lower level of myocardial metabolism became thinner, leading to alteration of regional wall stress and finally, the dilation of LV chamber. Such regional remodeling process was similar with the culprit vessel region after the attack of MI. Our work demonstrated that among MW indices, delta-WE, which was calculated as the absolute difference of WE between infarcted and non-infarcted territory, was independently associated with the incidence of LV remodeling. As revealed by previous studies, WE reflected the capacity of the myocardial to generate effective work based on hemodynamic parameters, oxygen consumption, and myocardial metabolism (25), and impaired global WE was associated with worse long-term survival after MI (26). Our findings suggest that imbalanced distribution of regional WE may serve as a trigger for adverse remodeling in post-infarct early LV remodeling, which may help risk stratification and provide new insight into mechanisms underlying cardiac remodeling in ischemic heart disease.

### Clinical Implications

LV remodeling after acute MI is an important risk factor for the development of heart failure and all-cause mortality. Our study proposed a novel and noninvasive echocardiographic index, delta-WE for predicting early remodeling. Despite relatively small sample size and short follow-up period, we have observed that patients who exhibited greater delta-WE at the acute phase were associated with higher incidence of heart failure during 3-month follow-up, indicating that more intensive management and early preventive strategy should be implicated in this subset. Our findings suggested that delta-WE might serve as an indicator for stratification of prognosis at the acute phase of MI. Our result also implies that innovative therapies targeting myocardial metabolic processes (e.g., sodium-glucose cotransporter-2 inhibitors and glucagon-like peptide-1 receptor agonists) (27) could be of help for reducing the risk of remodeling. Moreover, as inhomogeneous distribution of MW has been suggested as a potential trigger for the remodeling process in LV dyssynchrony (7, 8), delta-WE may also serve as a predictor for cardiac resynchronization therapy response.

### Limitations

First, this study is a single-center experience with a relatively small sample, and the predictive value of MW indices needs to be further confirmed by validation group. Second, it was noted that the imbalance of MW distribution, especially in patients with LV remodeling, still persisted at the end of 3-month follow-up. Since the process of post-infarct LV remodeling may continue for up to 1 year or longer, the association between such uneven work distribution and late remodeling needs to be investigated by long-term follow-up. Third, the number of patients with LVEF recovery in our cohort was relatively small due to short follow-up period. Further study is needed to explore the predictive value of myocardial work indices for LVEF recovery.

## Conclusion

After reperfused acute anterior STEMI, patients with LV remodeling presented with more inhomogeneous MW distribution. The absolute difference of WE between culprit and non-culprit territory at the acute phase is an independent predictor for early LV remodeling.

## Data Availability Statement

The raw data supporting the conclusions of this article will be made available by the authors, without undue reservation.

## Ethics Statement

The studies involving human participants were reviewed and approved by Ren Ji Hospital Ethics Committee + Ren Ji Hospital, School of Medicine, Shanghai Jiao Tong University. The patients/participants provided their written informed consent to participate in this study. Written informed consent was obtained from the individual(s) for the publication of any potentially identifiable images or data included in this article.

## Author Contributions

HZ and X-dS conceived and designed the study. WW analyzed myocardial work data and executed the statistical analysis. WW, HZ, and FW acquired clinical data. WW and HZ drafted the manuscript. SD and JP amended the manuscript. All authors contributed to the interpretation of the data and approved the final version of this manuscript.

## Funding

This study was supported by the National Key Research and Development Program of China [2018YFC1312802]; the Shanghai Jiao Tong University School of Medicine (DLY201804); the Shanghai ShenKang Hospital Development Center (SHDC12019X12); and the Shanghai Municipal Key Clinical Specialty (shslczdzk06204).

## Conflict of Interest

The authors declare that the research was conducted in the absence of any commercial or financial relationships that could be construed as a potential conflict of interest.

## Publisher's Note

All claims expressed in this article are solely those of the authors and do not necessarily represent those of their affiliated organizations, or those of the publisher, the editors and the reviewers. Any product that may be evaluated in this article, or claim that may be made by its manufacturer, is not guaranteed or endorsed by the publisher.

## Abbreviations

2D: Two-dimensional
AUC: Area under the curve
BNP: β-type natriuretic peptide
CMR: Cardiac magnetic resonance
DTE: Deceleration time of mitral E wave
GCW/CW: Global constructive work/constructive work
GLS: Global longitudinal strain
GWE/WE: Global work efficiency/work efficiency
GWI/WI: Global work index/work index
GWW/WW: Global wasted work/wasted work
ICC: Intraclass correlation coefficient
LAD: Left anterior descending coronary artery
LAV: left atrial volume
LV: Left ventricular
LVEDV: Left ventricular end-diastolic volume
LVEF: Left ventricular ejection fraction
LVESV: Left ventricular end-systolic volume
MI: Myocardial infarction
MW: Myocardial work
OR: Odds ratio
PCI: Percutaneous coronary intervention
ROC: Receiver operating curve
STE: Speckle-tracking echocardiography
STEMI: ST-segment elevation myocardial infarction
TIMI: Thrombolysis in myocardial infarction
TNI: Troponin I
WMSI: Wall motion score index.

## References

[1] van der Bijl P Abou R Goedemans L Gersh BJ Holmes DR Jr Ajmone Marsan N. Left ventricular post-infarct remodeling: implications for systolic function improvement and outcomes in the modern era. JACC Heart Fail. (2020) 8:131–40. 10.1016/j.jchf.2019.08.014 31838030

[2] Sutton MG Sharpe N. Left ventricular remodeling after myocardial infarction: pathophysiology and therapy. Circulation. (2000) 101:2981–8. 10.1161/01.CIR.101.25.2981 10869273

[3] Masci PG Ganame J Francone M Desmet W Lorenzoni V Iacucci I. Relationship between location and size of myocardial infarction and their reciprocal influences on post-infarction left ventricular remodelling. Eur Heart J. (2011) 32:1640–8. 10.1093/eurheartj/ehr064 21398642

[4] Bolognese L Neskovic AN Parodi G Cerisano G Buonamici P Santoro GM. Left ventricular remodeling after primary coronary angioplasty: patterns of left ventricular dilation and long-term prognostic implications. Circulation. (2002) 106:2351–7. 10.1161/01.CIR.0000036014.90197.FA 12403666

[5] Bauters C Dubois E Porouchani S Saloux E Fertin M de Groote P. Long-term prognostic impact of left ventricular remodeling after a first myocardial infarction in modern clinical practice. PLoS ONE. (2017) 12:e0188884. 10.1371/journal.pone.0188884 29176897 PMC5703528

[6] Burchfield JS Xie M Hill JA. Pathological ventricular remodeling: mechanisms: part 1 of 2. Circulation. (2013) 128:388–400. 10.1161/CIRCULATIONAHA.113.001878 23877061 PMC3801217

[7] Cvijic M Duchenne J Ünlü S Michalski B Aarones M Winter S. Timing of myocardial shortening determines left ventricular regional myocardial work and regional remodelling in hearts with conduction delays. Eur Heart J Cardiovasc Imaging. (2018) 19:941–9. 10.1093/ehjci/jex325 29272366

[8] Duchenne J Turco A Ünlü S Pagourelias ED Vunckx K Degtiarova G. Left ventricular remodeling results in homogenization of myocardial work distribution. Circ Arrhythm Electrophysiol. (2019) 12:e007224. 10.1161/CIRCEP.118.007224 31023060

[9] Bastos MB Burkhoff D Maly J Daemen J den Uil CA Ameloot K. Invasive left ventricle pressure-volume analysis: overview and practical clinical implications. Eur Heart J. (2020) 41:1286–97. 10.1093/eurheartj/ehz552 31435675 PMC7084193

[10] Russell K Eriksen M Aaberge L Wilhelmsen N Skulstad H Remme EW. A novel clinical method for quantification of regional left ventricular pressure-strain loop area: a non-invasive index of myocardial work. Eur Heart J. (2012) 33:724–33. 10.1093/eurheartj/ehs016 22315346 PMC3303715

[11] Manganaro R Marchetta S Dulgheru R Sugimoto T Tsugu T Ilardi F. Correlation between non-invasive myocardial work indices and main parameters of systolic and diastolic function: results from the EACVI NORRE study. Eur Heart J Cardiovasc Imaging. (2020) 21:533–41. 10.1093/ehjci/jez203 31408147

[12] Manganaro R Marchetta S Dulgheru R Sugimoto T Tsugu T Ilardi F. Global myocardial work is superior to global longitudinal strain to predict significant coronary artery disease in patients with normal left ventricular function and wall motion. J Am Soc Echocardiogr. (2019) 32:947–57. 10.1016/j.echo.2019.02.014 31043359

[13] Meimoun P Abdani S Stracchi V Elmkies F Boulanger J Botoro T. Usefulness of noninvasive myocardial work to predict left ventricular recovery and acute complications after acute anterior myocardial infarction treated by percutaneous coronary intervention. J Am Soc Echocardiogr. (2020) 33:1180–90. 10.1016/j.echo.2020.07.008 33010853

[14] Lustosa RP Fortuni F van der Bijl P Goedemans L El Mahdiui M Montero-Cabezas JM. Left ventricular myocardial work in the culprit vessel territory and impact on left ventricular remodelling in patients with ST-segment elevation myocardial infarction after primary percutaneous coronary intervention. Eur Heart J Cardiovasc Imaging. (2021) 22:339–47. 10.1093/ehjci/jeaa175 32642755

[15] Lustosa RP van der Bijl P El Mahdiui M Montero-Cabezas JM Kostyukevich MV Ajmone Marsan N. Noninvasive myocardial work indices 3 months after st-segment elevation myocardial infarction: prevalence and characteristics of patients with postinfarction cardiac remodeling. J Am Soc Echocardiogr. (2020) 33:1172–9. 10.1016/j.echo.2020.05.001 32651125

[16] Ibanez B James S Agewall S Antunes MJ Bucciarelli-Ducci C Bueno H. 2017 ESC Guidelines for the management of acute myocardial infarction in patients presenting with ST-segment elevation: The Task Force for the management of acute myocardial infarction in patients presenting with ST-segment elevation of the European Society of Cardiology (ESC). Eur Heart J. (2018) 39:119–77. 10.1093/eurheartj/ehx393 28886621

[17] Lang RM Badano LP Mor-Avi V Afilalo J Armstrong A Ernande L. Recommendations for cardiac chamber quantification by echocardiography in adults: an update from the American Society of Echocardiography and the European Association of Cardiovascular Imaging. J Am Soc Echocardiogr. (2015) 28:1–39.e14. 10.1016/j.echo.2014.10.003 25559473

[18] Meimoun P Abouth S Clerc J Elmkies F Martis S Luycx-Bore A. Usefulness of two-dimensional longitudinal strain pattern to predict left ventricular recovery and in-hospital complications after acute anterior myocardial infarction treated successfully by primary angioplasty. J Am Soc Echocardiogr. (2015) 28:1366–75. 10.1016/j.echo.2015.07.022 26341122

[19] Main ML Hannen MN Kusnetzky LL Martin JL Coggins TR Lanza P. Myocardial contrast echocardiographic estimates of infarct size predict likelihood of left ventricular remodeling after acute anterior wall myocardial infarction. J Am Soc Echocardiogr. (2006) 19:64–70. 10.1016/j.echo.2005.05.009 16423671

[20] Di Tano G Caretta G De Maria R Parolini M Bassi L Testa S. Galectin-3 predicts left ventricular remodelling after anterior-wall myocardial infarction treated by primary percutaneous coronary intervention. Heart. (2017) 103:71–7. 10.1136/heartjnl-2016-309673 27465055

[21] Boe E Russell K Eek C Eriksen M Remme EW Smiseth OA. Non-invasive myocardial work index identifies acute coronary occlusion in patients with non-ST-segment elevation-acute coronary syndrome. Eur Heart J Cardiovasc Imaging. (2015) 16:1247–55. 10.1093/ehjci/jev078 25851329

[22] Bogaert J Bosmans H Maes A Suetens P Marchal G Rademakers FE. Remote myocardial dysfunction after acute anterior myocardial infarction: impact of left ventricular shape on regional function: a magnetic resonance myocardial tagging study. J Am Coll Cardiol. (2000) 35:1525–34. 10.1016/S0735-1097(00)00601-X 10807456

[23] Hanratty CG Koyama Y Rasmussen HH Nelson GI Hansen PS Ward MR. Exaggeration of nonculprit stenosis during acute myocardial infarction: implications for immediate multivessel revascularization. J Am Coll Cardiol. (2002) 40:911–6. 10.1016/S0735-1097(02)02049-1 12225715

[24] Zhang Y Chan AK Yu CM Lam WW Yip GW Fung WH. Left ventricular systolic asynchrony after acute myocardial infarction in patients with narrow QRS complexes. Am Heart J. (2005) 149:497–503. 10.1016/j.ahj.2004.05.054 15864239

[25] Sörensen J Harms HJ Aalen JM Baron T Smiseth OA Flachskampf FA. Myocardial efficiency: a fundamental physiological concept on the verge of clinical impact. JACC Cardiovasc Imaging. (2020) 13:1564–76. 10.1016/j.jcmg.2019.08.030 31864979

[26] Lustosa RP Butcher SC van der Bijl P El Mahdiui M Montero-Cabezas JM Kostyukevich MV. Global Left Ventricular Myocardial Work Efficiency and Long-Term Prognosis in Patients After ST-Segment-Elevation Myocardial Infarction. Circ Cardiovasc Imaging. (2021) 14:e012072. 10.1161/CIRCIMAGING.120.012072 33653082

[27] Honka H Solis-Herrera C Triplitt C Norton L Butler J DeFronzo RA. Therapeutic manipulation of myocardial metabolism: JACC state-of-the-art review. J Am Coll Cardiol. (2021) 77:2022–39. 10.1016/j.jacc.2021.02.057 33888253 PMC8091273

